# Diabetes Mellitus Increased Mortality Rates More in Gender-Specific than in Nongender-Specific Cancer Patients: A Retrospective Study of 149,491 Patients

**DOI:** 10.1155/2012/701643

**Published:** 2012-06-03

**Authors:** Wen-Ko Chiou, Jawl-Shan Hwang, Kuang-Hung Hsu, Jen-Der Lin

**Affiliations:** ^1^Department of Industrial Design, Healthy Aging Research Center, Chang Gung University, Taoyuan 333, Taiwan; ^2^Division of Endocrinology and Metabolism, Department of Internal Medicine, Chang Gung Memorial Hospital, Chang Gung University, Taoyuan 333, Taiwan; ^3^Department of Health Care Management, Healthy Aging Research Center, Chang Gung University, Taoyuan 333, Taiwan

## Abstract

*Aims*. Hyperinsulinemia in overweight status, obesity, and type 2 diabetes mellitus (DM) is often accompanied by cancer. Gender is important in cancer epidemiology, clinical presentation, and response to therapy in different histological types of malignancy. Insufficient information is available concerning gender differences in DM with organ-specific and nonorgan-specific cancers. This study aimed to analyze gender differences in hospitalized cancer patients with or without type 2 DM. *Methods*. We retrospectively reviewed ten years of patients hospitalized in one institution, enrolling 36,457 female and 50,004 male cancer patients of which 5,992 females and 8,345 males were diagnosed as type 2 DM. *Results*. Statistically significant increases in incidence of type 2 DM were found in patients of both genders with pancreatic, liver, and urinary tract cancer. Increased incidence of type 2 DM was found in lung and hematologic malignancies in females and prostate cancer in males. Increases in mortality rates of females with type 2 DM (2.98%) were higher than those in males. DM increased mortality rates in gender-specific cancers from 1.91% (uterus, HR: 1.33) to 5.04% (ovary, HR: 1.49). *Conclusion*. Type 2 DM increased mortality of cancer patients of both genders, with higher increases in gender-specific than in nongender-specific cancers.

## 1. Introduction

Mortality rates for cancer with or without type 2 diabetes mellitus (DM) are much higher than in the general population worldwide [1–3]. Understanding the primary cause of this excess mortality is important in order to determine interventions to decrease mortality rates in these cases. The increased mortality has been attributed both to cancer treatment, underlying disease, or acute and chronic complications of DM, such as, renal and cardiovascular diseases (CVD). Previous reports have shown that DM may increase mortality rates of different cancers; in addition, we need to know that CVD as a predominate complication in type 2 DM may influence the results of treatment in different gender and age groups with type 2 DM [4–6]. Only a handful of recent reports address cause-specific mortality in population-based type 1 diabetes cohorts [7–12] and even fewer studies had long-term followup (15 years) [10, 11]. The aim of this study was to investigate gender differences in cancer patients with or without type 2 DM. Mortality causes are analyzed in different cancer histotypes.

## 2. Patients and Methods

### 2.1. Study Population

**Figure 1:**
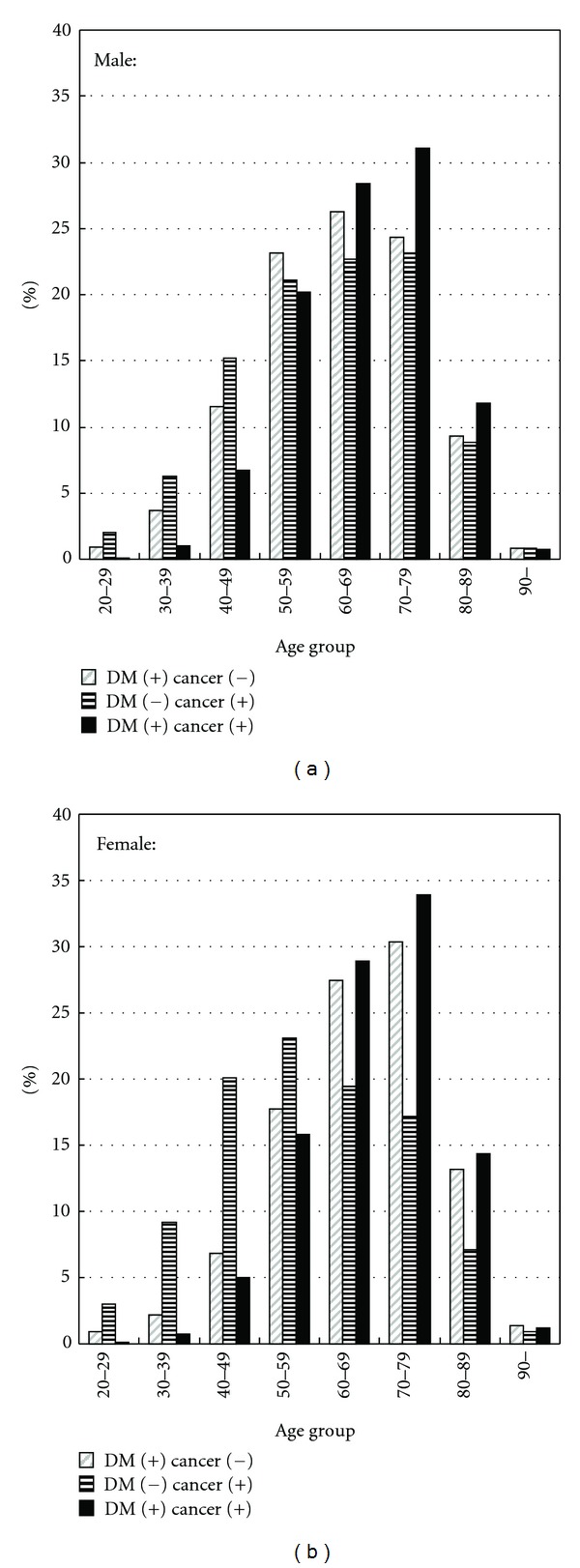
Percentage of cancer patients with and without type 2 DM and type 2 DM without cancer in different age groups of both genders.

Patients were identified through admission data from Chang Gung Memorial Hospital (CGMH) in Linkou, Taiwan, between January 2001 and December 2010. Patients were ≧20 years of age and were included if the indication for hospital admission was a diagnosis of type 2 DM or malignancy. Type 2 DM was defined as a fasting glucose level >126 mg/dL or a postprandial glucose level >200 mg/dL [13]; the diagnostic code of malignancy was defined as codes from 140–208.91 in the ICD-9 clinical modification format. A total of 149,491 cancer or type 2 DM patients included 81,564 males and 67,927 females. A total of 77,387 patients were diagnosed as type 2 DM. Most patients with type 2 DM were admitted for reasons other than DM. Disease codes for type 2 DM without cancer included infectious disease, cardiovascular episode, pulmonary diseases, accidence, and obstetric complications. In contrast, most cancer patients with or without DM were admitted for cancer diagnosis or therapy. Patients with type 1 DM were excluded from this study after ICD-9 code sorting.  Figure 1 illustrates age and gender distributions of hospitalized type 2 DM or cancer patients in this study. 

Death certificates from 2001 to 2010 were coded in ICD-9 [14]. Patients with malignant neoplasms required the diagnosis to be validated by at least two specialists based on examination of medical records, laboratory and imaging results, and histologic or cytologic analyses. Patients diagnosed with malignant neoplasms were categorized into different groups according to the anatomic organ system. All subjects were Chinese residents of Taiwan. The study was approved by the Institutional Review Board of CGMH.

### 2.2. Statistical Analysis

All data analyses were performed using SPSS (version 16.0; SPSS, Inc., Chicago, IL, USA) [15]. The incidence proportions were computed for various cancers and type 2 DM by gender and age. Logistic regression, adjusted for age and gender, was used to estimate the hazard ratios (HRs) and 95% confidence intervals (CIs) for associations between the DM mortality rate and the non-DM mortality rate of specific cancers. Chi-square tests were used to determine if the difference in frequency of a specific cancer between patients with and without DM was significant.

## 3. Results 

A total of 86,461 cases were diagnosed with malignancies including 14,337 patients with type 2 DM, including 36,457 females and 50,004 males. Among them, 5,992 (16.4%) females and 8,345 (16.7%) males (*P* > 0.05) were diagnosed as type 2 DM. The ratios of DM in female cancer patients were shown to be higher than in males after age 60 years.  Table 1 demonstrates the different histological patterns of cancer patients in both genders categorized in the DM and non-DM groups. The different histotypes with DM were variable, from 8.8% (thyroid cancer) to 31.5% (pancreas cancer). A statistically significant difference was found in the increased incidence of type 2 DM in patients with pancreas, liver, and urinary tract in both genders. In addition, increased incidence of type 2 DM of lung and hematologic malignancies in females and prostate cancer in males was found. A decreased incidence of DM was demonstrated in patients with thyroid and nasopharyngeal cancer in both genders. Furthermore, decreases in type 2 diabetes mellitus were noted in breast, cervical, and ovarian cancers in females and esophageal cancer in males. 

Table 2 shows HRs of mortality in cancer patients with DM and non-DM groups of different genders and histological types compared with type 2 DM patients without cancer. Significant increased mortality was shown in all pancreas, liver, lung, gastric, and hematologic cancers in both genders, and esophageal cancer in males in both DM and non-DM groups was higher than in DM without cancer. In contrast, in non-DM patients with prostate, colon, skin, and thyroid cancer in males and thyroid cancer in females without DM showed lower mortality rates than in DM without cancer. 

**Figure 2:**
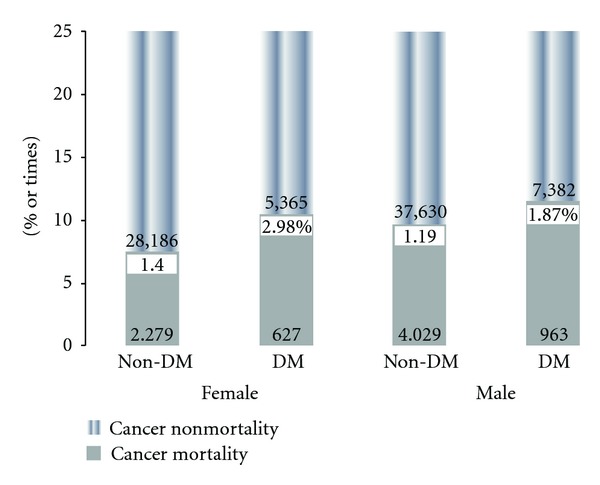
Total mortality rates of both genders diagnosed as cancer with and without type 2 DM.

Figure 2 illustrates total mortality rates of both genders diagnosed as cancer with and without type 2 DM in different age groups. DM with cancer in both genders demonstrated higher total mortality in all age groups except in those over age 90 years than in non-DM patients. Two peaks of mortality rates were shown in DM with cancer in ages younger than 40 years and in the 80–89-year-old age groups. Figure 2 illustrates case numbers and percentages of cancer with or without type 2 DM in both genders. In addition, increased mortality rates of DM patients with HR were described. Mortality rates of females with DM and cancer increased 2.98%, which was higher than that in males (1.87%) (HR: 1.40 in female, 1.19 in male; *P* < 0.01) (Figure 2).

**Figure 3:**
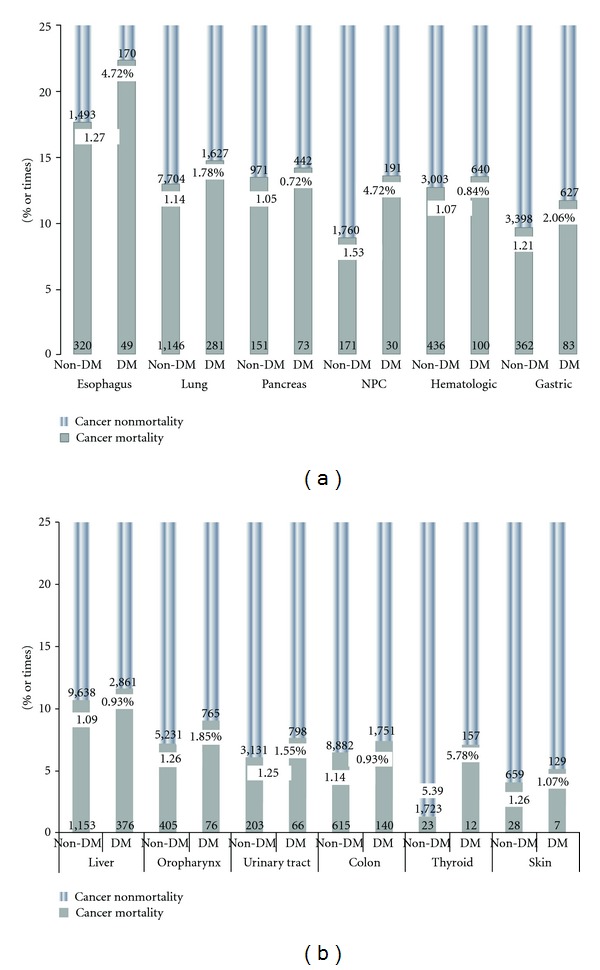
Mortality of individual nongender-specific cancer types in DM and non-DM groups.

**Figure 4:**
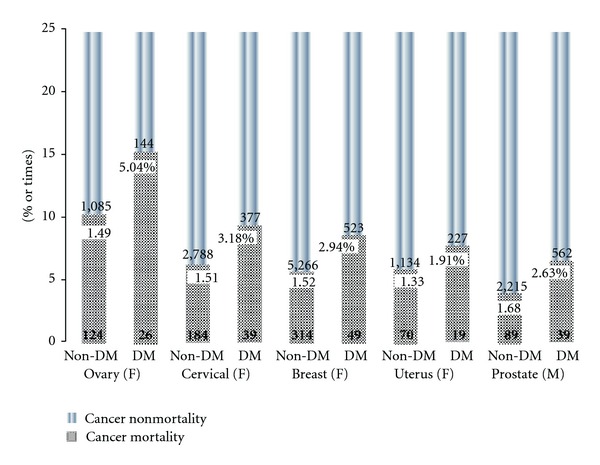
Mortality rates in gender-specific cancer with and without type 2 DM.

Comparison of individual cancers of nongender-specific types in DM and non-DM groups is illustrated in Figures 3(a) and 3(b). Type 2 DM increased mortality from 0.72% (pancreatic cancer, HR: 1.05) to 5.78% (thyroid cancer, HR 5.39) compared with non-DM patients. In addition, DM increased mortality rates in gender-specific cancer from 1.91% (uterus, HR: 1.33) to 5.04% (ovary, HR: 1.49) (Figure 4). In all patients, type 2 DM increased mortality in gender-specific cancer more than in nongender-specific cancer (2.69% versus 1.76%; HR: 1.46 versus 1.18; *P* < 0.01).

## 4. Discussion

The incidence of type 2 DM in hospitalized cancer patients in this study was slightly higher than in the general population in Taiwan; however, it was lower than in patients in previous studies with other comorbidities [16, 17]. In this cohort, age and gender showed characteristic patterns of type 2 DM with cancer. In the whole group, incidence of type 2 DM in cancer patients was not different between genders, but increased incidence of type 2 DM in females over age 60 was noted. These age and gender effects were partly due to the loss of estrogen protection in females during menopause as demonstrated in our previous study in healthy subjects [18].

In this study, incidence of type 2 DM in different cancer types was significantly lower than in cancer patients of both genders with nasopharyngeal and thyroid cancer. Recent studies showed that increased body mass index (BMI) may increase thyroid cancer incidence and risk [19, 20]. Otherwise, type 2 DM did not increase incidence of thyroid cancer in our recent study [21]. However, total mortality of thyroid cancer patients was increased in those with with type 2 DM. This result warrants further confirmation because most thyroid cancer patients have long-term followup. Until now, we have no information concerning type 2 DM incidence in patients with nasopharyngeal cancer. Contrary to our results, one study demonstrated that DM may result in worse disease-free survival of cancer patients than non-DM [22]. 

Oncogenesis is a multifactorial and multigenetic event. Gender and DM are known as important factors in the incidence and prognosis of many cancers. Compared with previous studies, mortality from site-specific malignancies is different in type 2 diabetic patients than in the non-DM population and a slight increase in the overall mortality from malignancies was observed in diabetic patients, achieving statistical significance in women but not in men [23]. In that study, type 2 DM had higher increases in mortality rates of female cancer patients than in male cancer patients.

In this study cohort of hospitalized patients, there were more males with nongender-specific cancers; however, females had a higher percentage of type 2 DM in nongender-specific cancer. The increased number of type 2 DM patients with cancer after age 60 years is partly due to loss of the estrogen protection effect of menopause; in addition, insulin-like growth factor 1 (IGF-1) may play a role. In a recent report, males had significantly higher serum IGF-1 levels than females in the age groups 18–24 and 50–69 but not in others [24]. Otherwise, other growth factors and environmental factors may play a role in oncogenesis, which needs to be further investigated.

In conclusion, type 2 DM increased overall mortality of cancer patients in both genders. A higher increment of mortality is found in females with type 2 DM with cancer than in males when compared with non-DM patients. Increased mortality rates of gender-specific cancer in patients with type 2 DM were higher than in nongender-specific cancer patients compared with those of non-DM cancer patients.

## Figures and Tables

**Table 1:** Number of cancer patients and DM (%) categorized in different gender and histological pattern.

Histology	Female	Male	Total	F/M ratio
	Total (DM) number [%]	Total (DM) number [%]	Total (DM) number [%]	
Pancreas	646 (220) [34.1%]*	991 (295) [29.8%]*	1,637 (515) [31.5%]	0.65
Liver	3,782 (1,030) [27.2%]*	10,246 (2,207) [21.5%]*	14,028 (3,237) [23.1%]	0.37
Urinary tract	1,565 (363) [23.2%]*	2,633 (501) [19.0%]*	4,198 (864) [20.6%]	0.59
Lung	3,600 (689) [19.1%]*	7,158 (1,219) [17.0%]	10,758 (1,908) [17.7%]	0.50
Gastric	1,639 (286) [17.4%]	2,831 (424) [15.0%]	4,470 (710) [15.9%]	0.58
Hematologic	1,844 (346) [18.8%]*	2,335 (394) [16.9%]	4,179 (740) [17.7%]	0.79
Colon	5,049 (865) [17.1%]	6,339 (1,026) [16.2%]	11,388 (1,891) [16.6%]	0.80
Skin	297 (55) [18.5%]	390 (81) [20.8%]	687 (136) [19.8%]	0.76
Oropharynx	631 (89) [14.1%]	5,846 (752) [12.9%]	6,477 (841) [13.0%]	0.11
NPC	601 (71) [11.8%]^*∧*^	1,551 (150) [9.7%]^*∧*^	2,152 (221) [10.3%]	0.39
Esophagus	135 (18) [13.3%]	1,897 (201) [10.6%]^*∧*^	2,032 (219) [10.8%]	0.07
Thyroid	1,460 (118) [8.1%]^*∧*^	455 (51) [11.2%]^*∧*^	1,915 (169) [8.8%]	3.21
Breast (F)	6,152 (572) [9.3%]^∧^	—	6,152 (572) [9.3%]	—
Cervical (F)	3,388 (416) [12.3%]^*∧*^	—	3,388 (416) [12.3%]	—
Ovary (F)		1,379 (170) [12.3]^*∧*^		—
	1,379 (170) [12.3]	—		
Uterus (F)	1,450 (246) [17.0%]	—	1,450 (246) [17.0%]	—
Prostate (M)	—	2,905 (601) [20.7%]*	2,905 (601) [20.7%]	—
Others	3,920 (658) [16.8%]	6,099 (792) [13.0%]	10,019 (1,450) [14.5%]	0.64

Total	36,457 (5,992) [16.4%]	50,004 (8,345) [16.7%]	86,461 (14,337) [16.6%]	0.73

**Table 2:** The hazard ratio concerning mortality with or without DM of admission cancer patients categorized in different cancer types and genders.

Cancers	Male				Female			
	Non-DM		DM		Non-DM		DM	
	HR	95% CI	HR	95% CI	HR	95% CI	HR	95% CI
Total cancer	2.50	[2.34, 2.66]	3.31	[2.78, 3.31]	2.27	[2.11, 2.45]	3.28	[2.96, 3.63]
Pancreas	2.19	[1.77, 2.69]	2.00	[1.44, 2.77]	1.91	[1.40, 2.61]	2.66	[1.82, 3.89]
Liver	1.65	[1.53, 1.78]	1.75	[1.53, 2.00]	1.63	[1.42, 1.87]	2.05	[1.68, 2.45]
Uterus (F)					0.99	[0.78, 1.26]	1.34	[0.84, 2.15]
Urinary tract	0.86	[0.72, 1.02]	0.95	[0.68, 1.33]	0.84	[0.65, 1.09]	1.39	[0.95, 2.04]
Lung	1.99	[1.84, 2.16]	2.34	[1.99, 2.74]	2.41	[2.15, 2.71]	2.75	[2.21, 3.42]
Gastric	1.32	[1.15, 1.51]	1.73	[1.30, 2.31]	1.67	[1.39, 2.02]	1.91	[1.31, 2.79]
Prostate (M)	0.47	[0.38, 0.59]	0.82	[0.59, 1.13]				
Hematologic	1.94	[1.70, 2.22]	2.16	[1.63, 2.85]	2.13	[1.81, 2.50]	2.22	[1.60, 3.08]
Colon	0.78	[0.70, 0.88]	1.00	[0.79, 1.25]	1.18	[1.05, 1.34]	1.21	[0.93, 1.57]
Skin	0.57	[0.33, 0.97]	0.94	[0.41, 2.17]	0.48	[0.22, 1.01]	0.30	[0.04, 2.14]
Cervical (F)					1.07	[0.91, 1.24]	1.67	[1.20, 2.33]
Oropharynx	0.96	[0.86, 1.07]	1.21	[0.94, 1.55]	0.84	[0.57, 1.24]	1.37	[0.63, 2.96]
Ovary (F)					1.87	[1.55, 2.26]	2.93	[1.93, 4.45]
NPC	1.18	[0.98, 1.42]	1.54	[0.93, 2.55]	1.53	[1.13, 2.08]	3.62	[1.98, 6.61]
Breast (F)					0.96	[0.85, 1.08]	1.50	[1.12, 2.01]
Esophagus	2.71	[2.39, 3.08]	3.79	[2.73, 5.26]	2.55	[1.50, 4.32]	2.01	[0.46, 8.74]
Thyroid	0.24	[0.12, 0.49]	1.01	[0.37, 2.82]	0.18	[0.11, 0.30]	1.14	[0.55, 2.33]
